# Supplementary material for: Deep Mutational Scanning of FDX1 Identifies Key Structural Determinants of Lipoylation and Cuproptosis
Source: Nat Commun. 2025 Dec 21;17:1112. doi: 10.1038/s41467-025-67869-0 (PMC12855868; doi:10.1038/s41467-025-67869-0)
Supplement: Supplementary file 1 — Supplementary Information [file 41467_2025_67869_MOESM1_ESM.pdf]

Supplementary Materials for

Deep Mutational Scanning of FDX1 Identifies Key Structural Determinants of Lipoylation and Cuproptosis

Hsiao & Warui *et al.*

Correspondence to Peter Tsvetkov: ptsvetko@bidmc.harvard.edu

This PDF file includes:

Supplementary Figures 1 to 3

Supplementary Figure 1

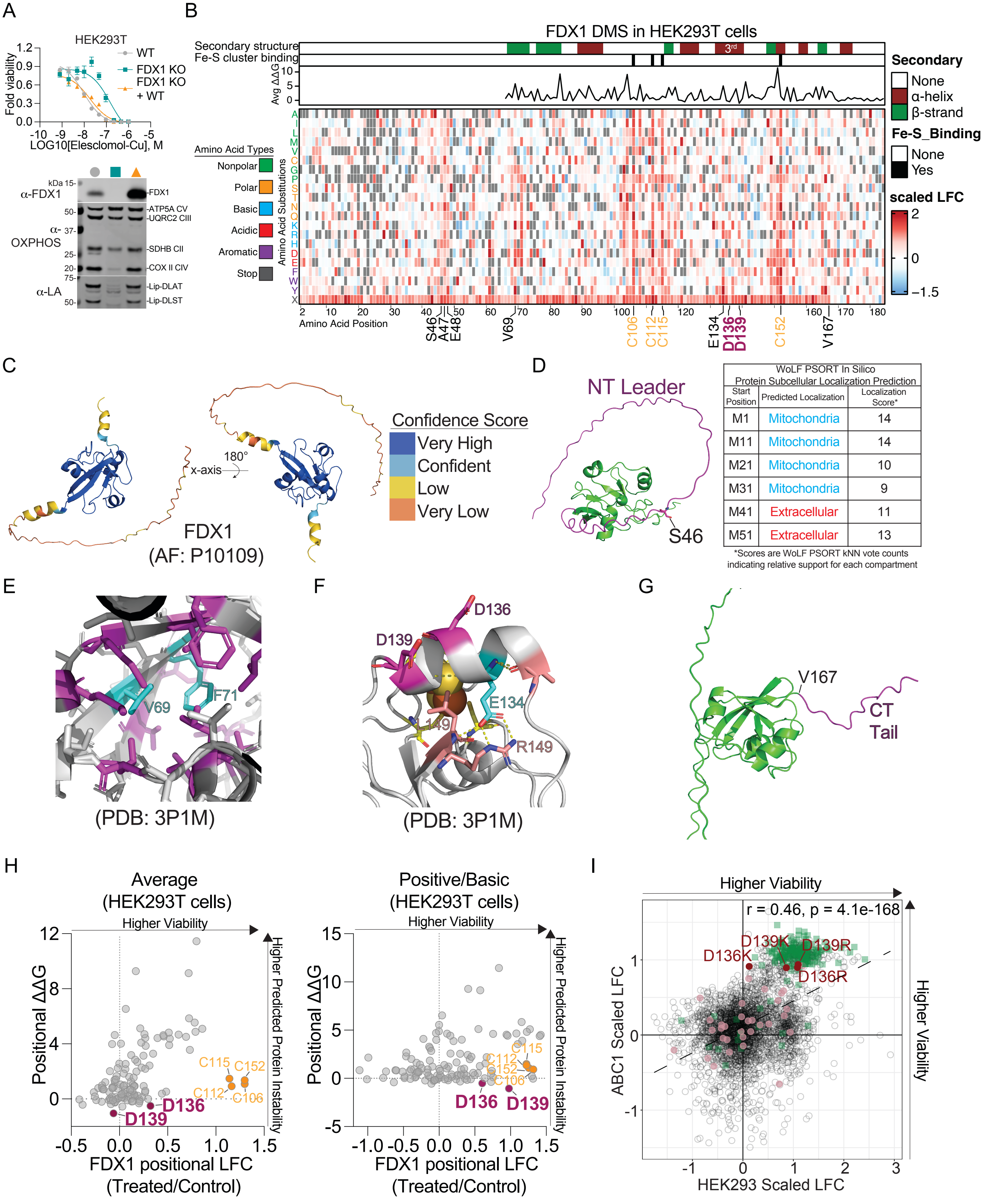

WoLF PSORT In Silico Protein Subcellular Localization Prediction		
Start Position	Predicted Localization	Localization Score*
M1	Mitochondria	14
M11	Mitochondria	14
M21	Mitochondria	10
M31	Mitochondria	9
M41	Extracellular	11
M51	Extracellular	13

*Scores are WoLF PSORT kNN vote counts indicating relative support for each compartment

Supplementary Figure 1 (related to Figure 1&2). DMS screen in HEK293Ts reveals D136 and D139 as key residues on FDX1 regulating cuproptosis. (**A-B**) Same as with Figure 1B and Figure 2A, respectively, but in HEK293Ts instead of ABC1s. Immunoblots were performed with the indicated antibodies on the corresponding HEK293T cell lines used for calibration. Viability data is presented as mean ± SD of four biological replicates in (**A**). (**C**) Structure of full-length FDX1 retrieved from AlphaFold Database (DB). Confidence scores are defined as the following: very high = pIDDT>90; confident = 90>pIDDT>70; low = 70>pIDDT>50; very low = pIDDT<50. (**D**) Left, same predicted structure in (**C**) but with substitution-intolerant residue S46 indicated. The disordered region is colored in magenta. Right, summary of the results of a WoLF PSORT (https://wolfpsort.hgc.jp/) protein subcellular location prediction performed on sequentially truncated FDX1 from the N-terminus. (**E-F**) Structural models based on the crystal structure of FDX1 (PDB: 3P1M), highlighting substitution-intolerant residues V69–F71 (**E**) and E134 (**F**). (**G**) Same predicted structure in (**C**) but with the C-terminal disordered tail colored in magenta. V167, the last residue on the C-terminus if mutated that severely impairs impact FDX1 function, is indicated. (**H**) Same as with Figure 2B, but in HEK293Ts instead of ABC1s. (**I**) Scatterplot indicates each FDX1 mutant's LFC in viability in HEK293Ts (y-axis) and in ABC1s (x-axis). Mutations of interest are indicated. LFC, log fold change. The correlation coefficient and the p-value are indicated. Colored in maroon are the positively charged substitutions of D136/139. Colored in pink are the other substitutions of D136/139, and colored in green are the nonsense mutations of all positions.

Supplementary Figure 2

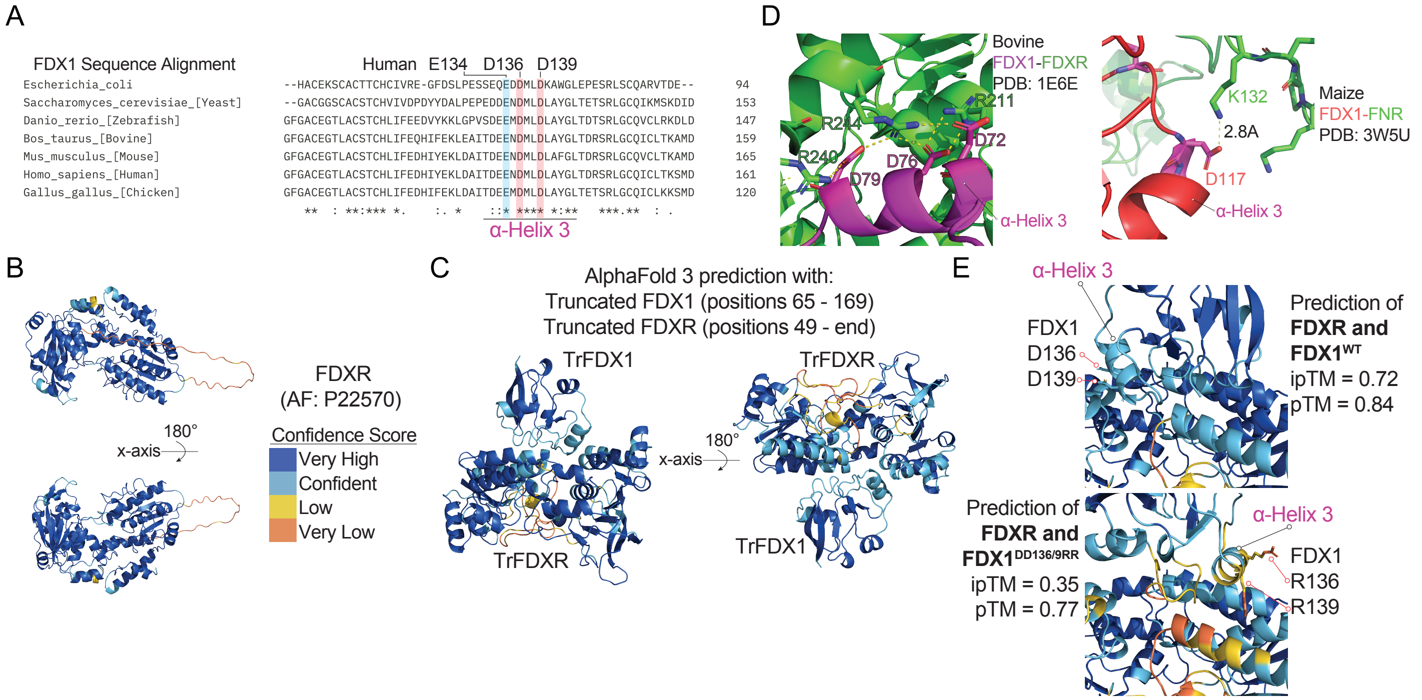

Supplementary Figure 2 (related to Figure 4). Structural models of the interaction between FDX1 and FDXR. (**A**) Multiple sequence alignment (using Clustal Omega) of the regions surrounding the third alpha helix in FDX1 from the indicated species. Asterisk, complete conservation; colon, strong conservation; period, weak conservation. The two completely conserved aspartic acids of interest (human D136 and D139) are highlighted in light red, and human E135 in light blue. The region that constitutes α-Helix 3 (colored green) is underscored. (**B**) Structure of full-length FDXR retrieved from AlphaFold DB. (**C**) AlphaFold3 prediction of truncated FDX1 and truncated FDXR, trimmed of its flanking disordered regions. (**D**) Left panel, crystal structure of *Bos taurus* (bovine) FDX1 in a cross-linked complex with FDXR. Right panel, crystal structure of *Zea mays* (maize) FDX1 in a cross-linked complex with Ferredoxin-NADP+ reductase (FNR). PDB codes are indicated. (**E**) Alphafold 3 predictions between truncated FDXR and truncated FDX1 (top) or truncated DD136/139RR FDX1 (bottom). In (**B-C**) and (**E**), confidence scores are defined as the following: very high = pIDDT>90; confident = 90>pIDDT>70; low = 70>pIDDT>50; very low = pIDDT<50.

Supplementary Figure 3

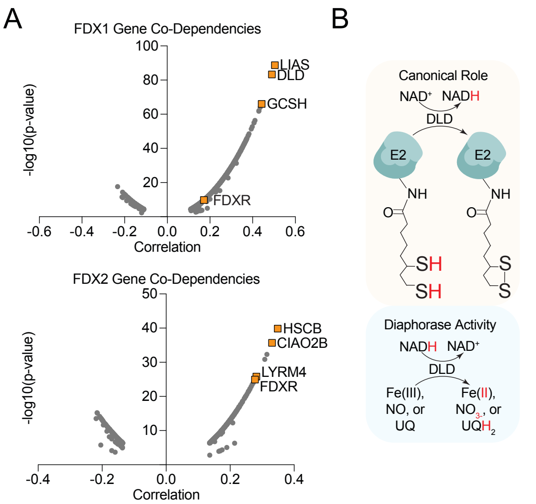

C

Selected Foldseek Hits (Analysis Restricted to Mammalia)				
	Name	Species	Alphafold/Uniprot	Score (E-Value)
Query:	NADPH:adrenodoxin oxidoreductase, mitochondrial (FDXR)	Human	P22570	0.00E+00
Targets:	NADPH:adrenodoxin oxidoreductase, mitochondrial (FDXR)	Rat	P56522	9.28E-99
	NADPH:adrenodoxin oxidoreductase, mitochondrial (FDXR)	Mouse	Q61578	1.23E-97
	Dihydropyrimidine dehydrogenase [NADP(+)] (DPYD)	Human	Q12882	3.91E-15
	Dihydrolipoyl dehydrogenase, mitochondrial (DLD)	Human	P09622	8.06E-12
	Dihydrolipoyl dehydrogenase, mitochondrial (DLD)	Mouse	O08749	1.39E-12
	Dihydrolipoyl dehydrogenase, mitochondrial (DLD)	Rat	Q6P6R2	2.19E-11

Selected DALI Server Hits (Analysis Restricted to Humans)				
	Name	Species	Alphafold/Uniprot	Score (Z-Score)
Query:	NADPH:adrenodoxin oxidoreductase, mitochondrial (FDXR)	Human	P22570	58.6
Top 5 Targets	Dihydrolipoyl dehydrogenase, mitochondrial (DLD)		P09622	20.2
	Dihydropyrimidine dehydrogenase (DPYD)		Q12882	16.9
	Ferroptosis suppressor protein 1 (FSP1/AIFM2)		Q9BRQ8	14.8
	Apoptosis-inducing factor 1, mitochondrial (AIFM1)		O95831	14.2
	Oxidative stress-induced growth inhibitor 1 (OSGIN1)		Q9UJX0	14

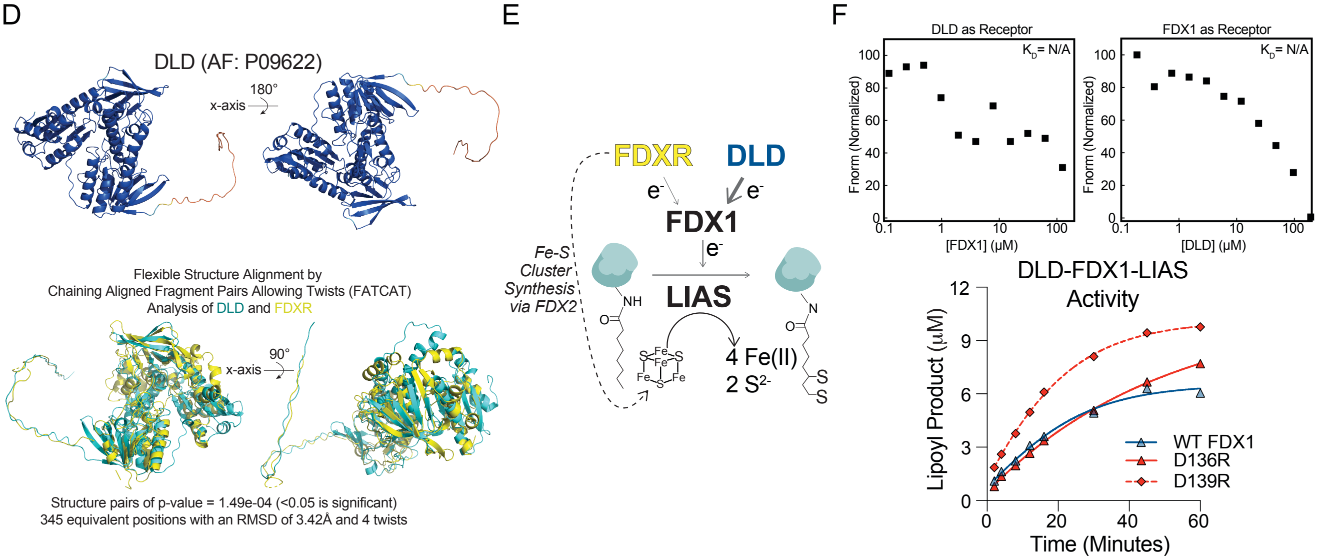

Supplementary Figure 3 (related to Figure 5). Supporting evidence that DLD can function as an FDX1 reductase. (**A**) The genetic co-dependencies of FDX1 (top) and FDX2 (bottom). LIAS and GCSH participate in lipoic acid biosynthesis, while HSCB, CIAO2B, and LYRM4 participate in iron-sulfur cluster biosynthesis (see methods for details). (**B**) Two roles of DLD are indicated. NAD, nicotinamide adenine dinucleotide. Fe, iron. NO, nitric oxide. UQ, ubiquinol. (**C**) Top results of Foldseek and DALI analyses using FDXR as the query and the Alphafold database as targets. Foldseek E-values less than 10^{-7} and DALI Z-scores more than 2 are considered highly significant with strong structural similarity. (**D**) Top panel, structure of full-length DLD retrieved from AlphaFold DB. Bottom panel, FATCAT analysis aligning AlphaFold predicted DLD and FDXR (**Supplementary Figure 2B**) are shown to indicate their structural similarity. RMSD, root mean square deviation. (**E**) Molecular model depicting FDXR's contribution towards Fe-S cluster synthesis, which confounds its role as a direct electron donor to FDX1. (**F**) Top panels, dianthus binding affinity tests of serially diluted WT FDX1 with red-NHS labelled 50 nM DLD as receptor (left panel) and serially diluted DLD with Red-tris NTA labelled 50 nM FDX1 as receptor (right panel). Bottom panel, in vitro DLD- and LIAS-mediated lipoylation activity assay that compares FDX1 WT versus the mutants, data represents the mean of three replicates.
